# Congenital systemic venous return anomalies to the right atrium review

**DOI:** 10.1186/s13244-019-0802-y

**Published:** 2019-12-04

**Authors:** João Diogo Oliveira, Isa Martins

**Affiliations:** Radiology Department, Centro Hospitalar Lisboa Ocidental E.P.E., Estrada do Forte do Alto do Duque, 1449-005 Lisboa, Portugal

**Keywords:** Vena cava (superior), Vena cava (inferior), Venae cavae, Azygos vein, Congenital abnormalities, Incidental findings

## Abstract

Congenital anomalies of the systemic venous return to the right atrium are rare and stem from variations in the embryogenesis of the venous system. They are usually asymptomatic, and such the major clinical significance of their recognition is to prevent misdiagnosis, in addition to some having technical implications on invasive procedures.

Typically, the venous blood from the upper half of the body is carried by the right-sided, superior vena cava (SVC), and some common congenital abnormalities found are persistent left SVC, SVC duplication, anomalous drainage of the brachiocephalic veins, or interruption of the SVC. The venous blood from the lower body is carried by the right-sided, inferior vena cava (IVC), and some common congenital abnormalities found are left-sided IVC, IVC duplication, the absence of IVC (total or just the infrarenal segment), and azygos continuation of the IVC. The azygos system of veins, running up the side of the thoracic vertebral column, connects both systems and can provide an alternative path to the right atrium when either of the *venae cavae* is absent. Other associated azygos-hemiazygos system anomalies are the azygos lobe and variable configuration of the azygos and hemiazygos veins.

Such anomalies are reviewed with particular respect to their embryology and imagiological presentation, as knowledge of the normal anatomy and the most common congenital anomalies of the systemic venous return by a radiologist is important, being incidentally found.

## Key points

- Congenital anomalies of the systemic venous return are rare.
- They are the result of complex deviations in the persistence/regression of segments of the primitive venous network.
- There can be anatomic variations of the superior vena cava, the inferior vena cava, and the azygos-hemiazygos venous system.
- The azygos-hemiazygos system can provide an alternative path to the right atrium when either of the *venae cavae* is absent/interrupted.
- The major clinical significance of their recognition is to prevent misdiagnosis yet can have implications on invasive procedures.

## Background

Knowledge of the normal anatomy and the most common congenital abnormalities of the systemic venous return by a radiologist is important as although rare, these are incidentally found.

**Fig. 1:**
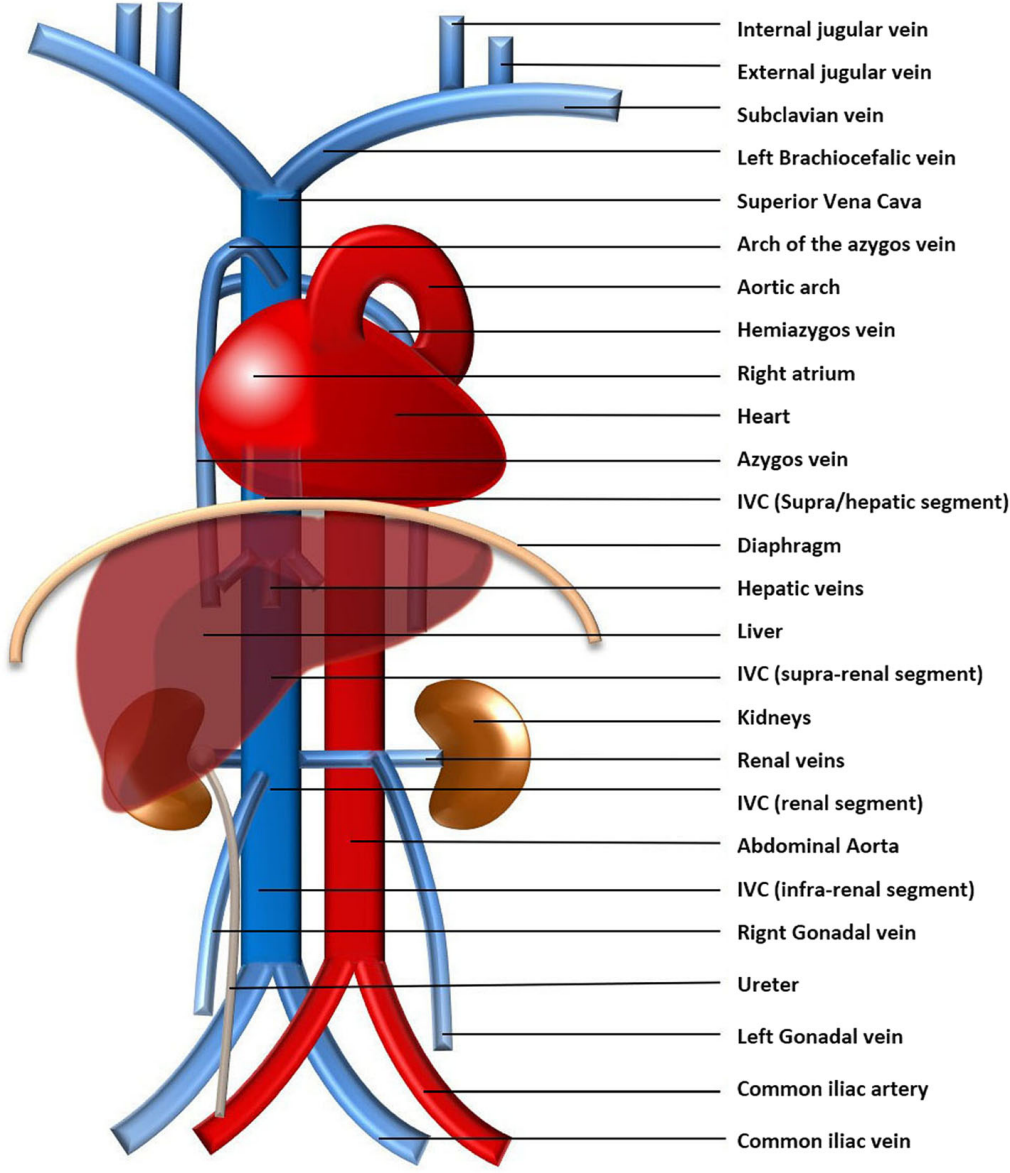
Normal systemic venous system schematic. The typical systemic venous system consisting of the right-sided superior vena cava, result of the confluence of the brachiocephalic veins, and the right-sided inferior vena cava (IVC), formed by the confluence of the common iliac veins, which respectively, drain the venous blood from the upper and lower body to the right atrium. Due to its particular embryology and relationship with the abdominal organs, the IVC can be subdivided in different segments (suprahepatic, hepatic, suprarenal, renal, infrarenal segments) and is asymmetric, with the gonadal and suprarenal veins draining directly into it on the right but to the left renal vein, instead, on the left. The azygos-hemiazygos venous system, running on the side of the vertebral column, drains the thoracic wall and superior lumbar region into the superior vena cava via the arch of the azygos vein

Typically, the venous blood from the upper half of the body is carried by the right-sided superior vena cava (SVC) resulting from the confluence of the brachiocephalic veins. The blood from the lower body drains commonly through the right-sided inferior vena cava (IVC), formed by the confluence of the common iliac veins (Fig. 1). The azygos-hemiazygos venous system, running up the side of the vertebral column, may connect both systems and provide an alternative path to the right atrium when either of the *venae cavae* is interrupted.

**Fig. 2:**
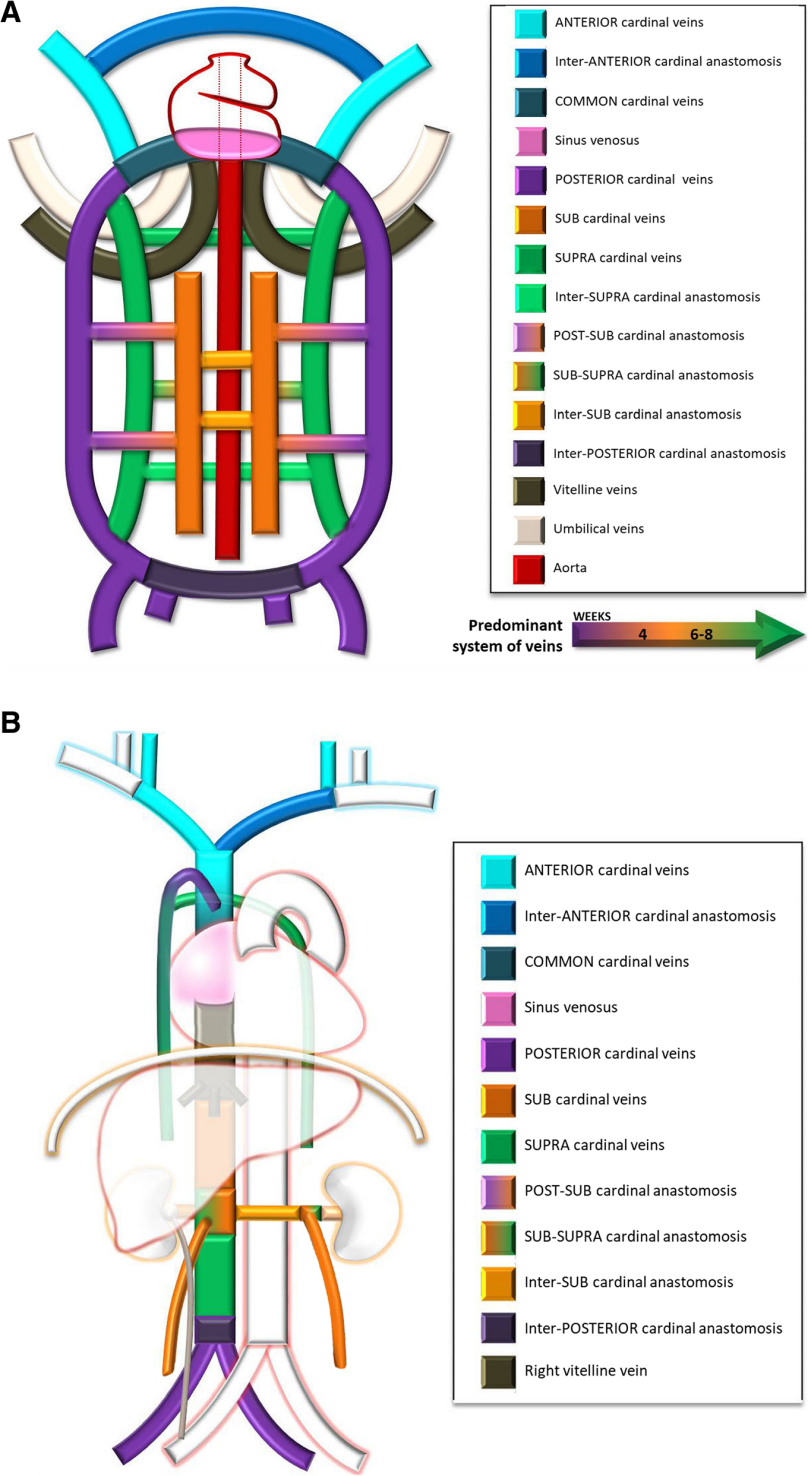
**a** Systemic venous system development composite schematic. The non-contemporary primitive venous network consisting of symmetrically paired venous trunks and interconnecting anastomosis, of which a dorsal systemic system, comprising of the *anterior* (light blue) and *posterior* cardinal (purple) veins, the *sub*cardinal veins (orange), and the *supra*cardinal veins (green), each predominating subsequently and which carries all the intraembryonic blood, and a double nutritional network, comprising the vitelline (brown) and umbilical veins (white). The precursor of the atrium, then known as the *sinus venosus*, is seen in (pink). **b** Normal systemic venous system embryology schematic. The normal end result of the complex deviations in the primitive venous network, resulting in composed single major trunks on the right side and regression of the left-sided vessels: the SVC being formed by the right *anterior* cardinal vein (light blue); the left brachiocephalic venous trunk by the inter-*anterior* cardinal anastomosis (dark blue); the suprahepatic and hepatic segments of the IVC by the right vitelline vein and right vitelline-*sub*cardinal anastomosis (brown); the suprarenal, infrarenal, and renal segments of the IVC respectively by the right *sub*cardinal vein (orange), the right *supra*cardinal vein (green), and the anastomosis between them; the terminal IVC and common iliac veins by the *posterior* cardinal veins; and the azygos-hemiazygos venous system from the *supra*cardinal veins, with the azygos arch resulting from an upper segment of the right *posterior* cardinal vein

Variations to the common anatomy are the result of complex deviations in the persistence and regression of segments of the primitive venous network, which in primordial stages of fetal development consists of a dorsal systemic network (carrying all the intraembryonic blood, and formed by the paired anterior and posterior cardinal veins which through the common cardinal veins reach the *sinus venosus*, the precursor of the atrium) and a double nutritional network (the vitelline, which carries the extraembryonic blood from the yolk sac and the umbilico-allantoic system carrying oxygenated blood from the placenta). Initially, these are symmetrically paired (Fig. 2a) but, following a succession of cross and transverse anastomoses, are converted into single major trunks on the right side, whereas the left-sided vessels regresses and are normally obliterated (Fig. 2b) [1, 2]

These anomalies are usually asymptomatic, and such the major clinical significance of their recognition is to prevent misdiagnosis as adenopathy and in a preoperative setting [3, 4].

Anomalous drainage to the left atrium can also occur but is usually associated with some sort of congenital heart disease, being rare if the heart is normal [5, 6].

## Main text

### Anomalies from the upper body venous return

#### Anatomy

The Superior Vena Cava (SVC) is a large valveless venous trunk on the right side of the mediastinum, resulting from the confluence of the brachiocephalic veins at the level of the first costal cartilage. It drains blood from the upper half of the body, except the heart, to the right atrium at the level of the right third costal cartilage [7].

#### Embryogenesis

**Fig. 3:**
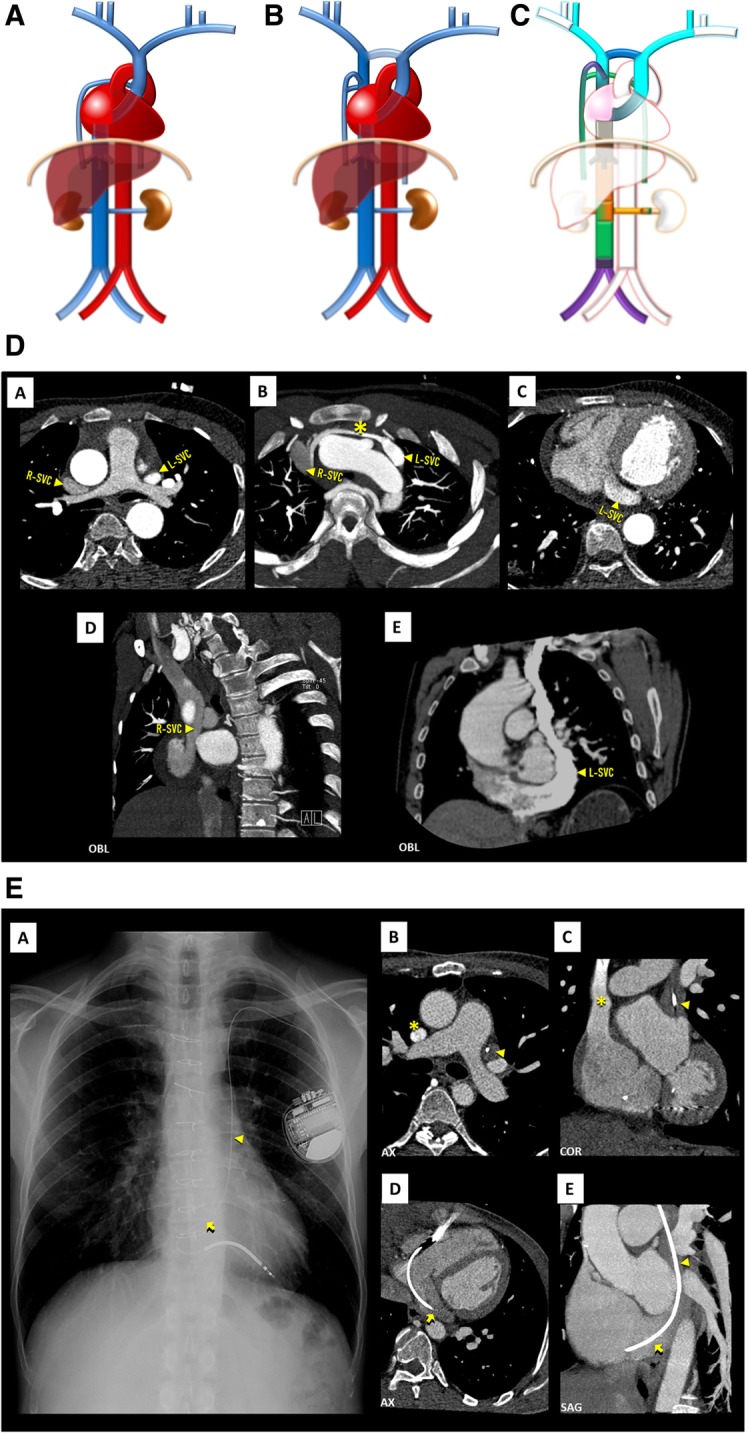
**a** Left SVC schematic. A single left SVC, with its usual course lateral to the aortic arch and drainage into the coronary sinus. **b** Double SVC schematic. The most frequent case of left SVC persistence, in which there are two SVC, each running on one side of the mediastinum, the right draining normally and the left one draining typically to the coronary sinus. These may or, more frequently, may not be connected by the left brachiocephalic vein. **c** Double SVC embryology schematic. Double SVC resulting from the persistence of the left *anterior* cardinal vein, here connected by the inter-*anterior* cardinal anastomosis (dark blue), precursor of the left brachiocephalic vein. If, in addition, there was regression of the right *anterior* cardinal vein, the end result would be a single left SVC. **d** Double SVC imaging. Contrast-enhanced CT showing double SVC, with a left-sided SVC (L-SVC) along the left side of the mediastinum [A] and draining into an enlarged coronary sinus [C–E], connected to a normal right-sided SVC (R-SVC) by the left brachiocephalic vein (asterisk) [B]. **e** Persistent left SVC eventuated by left-sided implantable cardioverter defibrillator leads. Chest X-ray [A] and contrast-enhanced CT scan [B–D] showing persistent left SVC (triangle) eventuated by a left-sided implantable cardioverter defibrillator lead, entering the right atrium via the coronary sinus (arrow)

It develops at a later time of fetal development than the IVC and has a relatively simple development compared to it. All begins with the fusion of the venous plexuses of the upper limbs culminating in the subclavian veins, which end up opening into the anterior cardinal veins. Then on the eighth week of development, a large anterior cardinal veins anastomosis, derived from the thyroid and thymic veins, communicates the left anterior cardinal vein to the right one, resulting in the future left brachiocephalic venous trunk. The left anterior cardinal vein and left common cardinal vein regresses, persisting only in short segments, which form, respectively, the left superior intercostal vein, and the coronary sinus (Fig. 2). From the persistence of the left anterior cardinal vein results a double SVC, and if, in addition, there is regression of the right cardinal vein, ultimately there is a single left SVC (Fig. 3c) [1, 2].

#### Anomalies

Persistent left SVC (Fig. 3a)/SVC duplication (Fig. 3b): A persistent left SVC, in most cases as a component of a double SVC (Fig. 3d), is an incidental finding in < 0.5% of the general population, occurring most frequently with congenital heart disease, where the incidence can reach 4% [3, 7]. In the absence of congenital heart disease, the left component of a double SVC or the single left SVC, usually drains into the coronary sinus, entering the pericardium at the posterior atrioventricular groove, with a course lateral to the aortic arch, passing in front of the pulmonary hilum. On SVC duplication, most frequently, the right trunk is smaller than the left and the left brachiocephalic vein is absent, but there may be a left brachiocephalic vein connecting both SVCs. In conventional radiography, it can be identified by a focal widening of the superior left cardiomediastinal contour, adjacent to the aortic knob [7], or most commonly when traversed by an intravenous catheter/electrode leads (Fig. 3e).

**Fig. 4:**
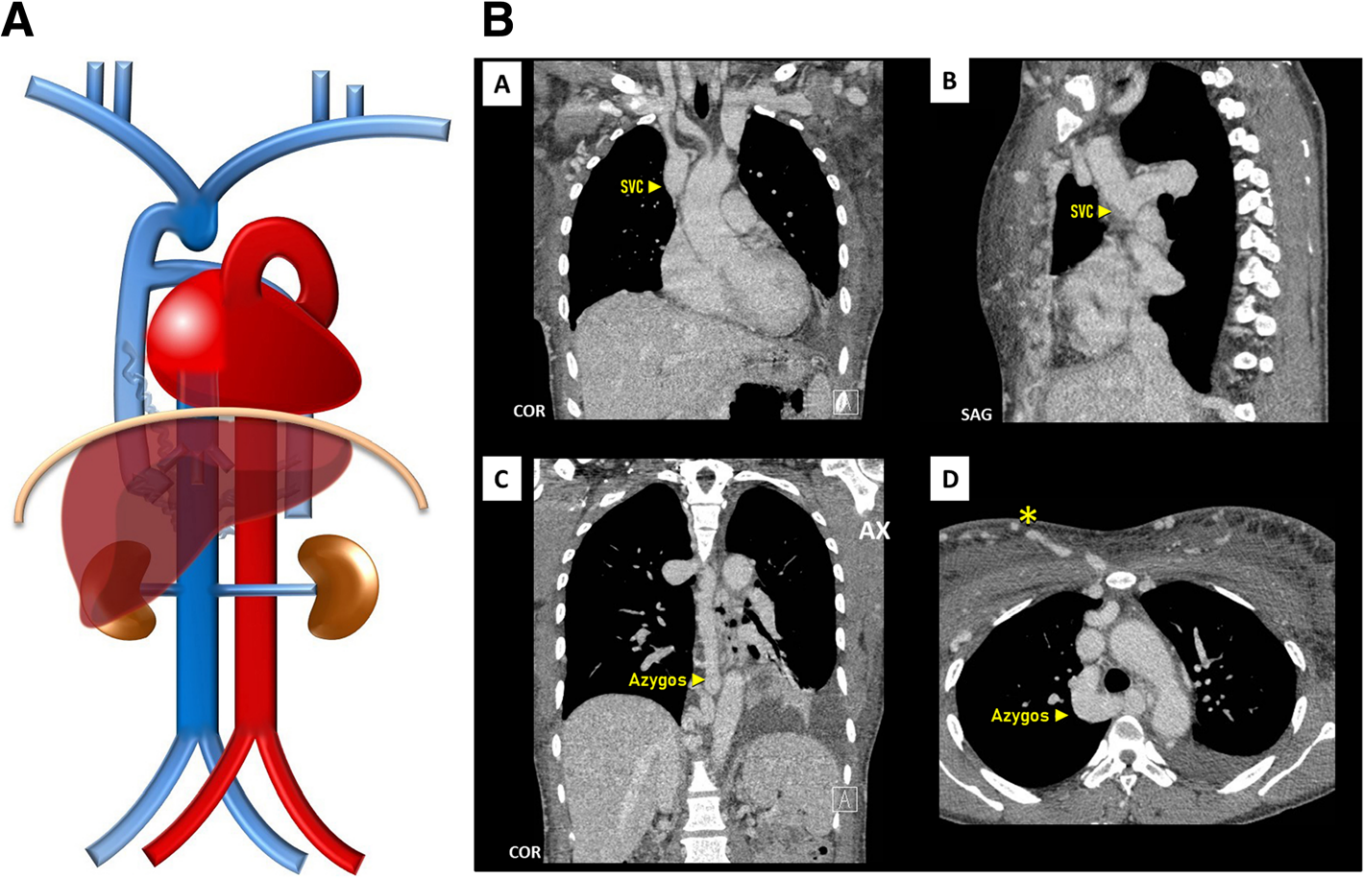
**a** Interrupted SVC schematic. In an absence of the SVC, the venous blood from the upper body drains into the persistent azygo-hemiazygos system of veins subdiaphragmatically, with prominent collateral vessels that allow a sufficient venous return by cavo-caval anastomoses. **b** Interrupted SVC imaging contrast-enhanced CT showing an interruption of the SVC [A, B] with exuberant collateral circulation by the azygos-hemiazygos venous system [C, D] and collaterals of the anterior chest wall (asterisk)

Interruption of the SVC (Fig. 4a/b): Absence of the right SVC is almost always related with persistence of a left SVC, so total absence of SVC is a very rare anomaly, often found with other congenital cardiac anomalies and/or conduction abnormalities. The superior systemic veins drain into persistent azygos veins subdiaphragmatically, with prominent collateral vessels that allow a sufficient venous return by cavo-caval anastomoses. The patient may be asymptomatic, but many suffer from SVC syndrome. It also can complicate the placement of intravenous catheters and transvenous pacemaker electrode leads [3].

**Fig. 5:**
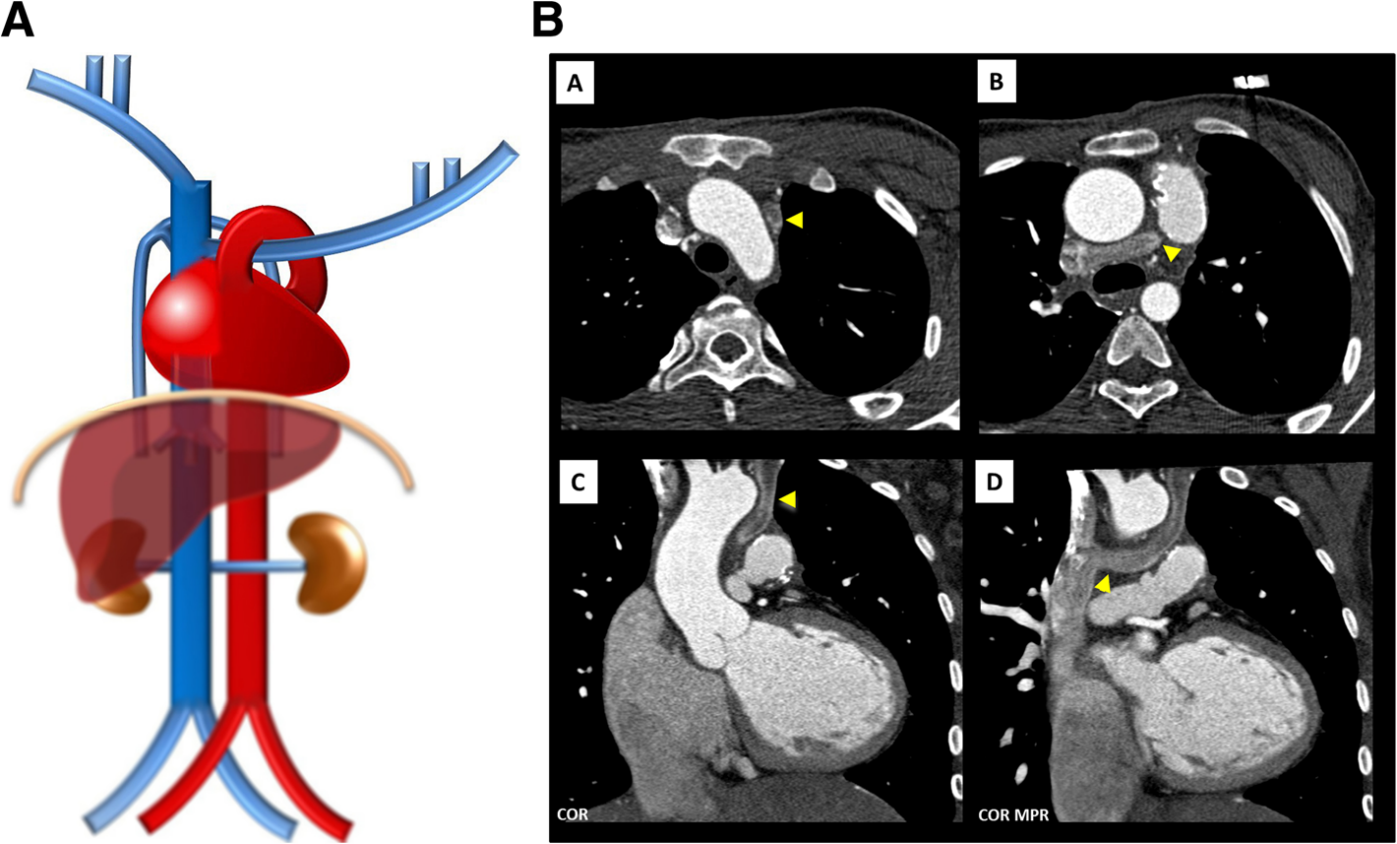
**a** Anomalous course of the left brachiocephalic vein schematic. Anomalous course of the left brachiocephalic vein, crossing dorsal to the ascending aorta, beneath the aortic arch, ending on the SVC caudal to the azygos vein. **b** Anomalous course of the left brachiocephalic vein imaging. Contrast-enhanced CT [A, B—axial plane, C, D—coronal plane] showing an anomalous course of the left brachiocephalic vein (triangle) draining into the SVC dorsal to the ascending aorta with a course in the aortopulmonary window

Anomalous course of the brachiocephalic veins (Fig. 5a/b): The left brachiocephalic vein occasionally follows a deviant course, one of which consist of crossing dorsal to the ascending aorta, beneath the aortic arch, ending on the SVC caudal to the azygos vein. It is usually associated with congenital heart disease [3].

Isolated anomalies of the SVC are rare and consist of low insertion into the right atrium or aneurysmal dilatation [5, 6].

### Anomalies from the lower body venous return

#### Anatomy

The inferior vena cava (IVC) results from the confluence of the two common iliac veins at about the fifth lumbar vertebra. It drains the abdomen, pelvis, and lowers limbs to the right atrium, crossing the diaphragm through the vena cava foramen, having a short intra-thoracic course. In the abdominal cavity, it has a retroperitoneal course, running on the right side of the spine and the aorta. Due to not being a midline structure, the drainage is asymmetric, with the gonadal and suprarenal veins on the right side draining directly into the IVC, but the ones on the left side to the left renal vein instead [8].

#### Embryogenesis

Normal IVC development occurs between the sixth and eighth weeks of embryological life and is more complex, with contribution of many embryological veins, chronologically, the posterior cardinal, the subcardinal, and the supracardinal veins (Fig. 2a), each predominating temporarily, then regressing, remaining only partly in the final venous system. The definitive IVC is such composed of diverse segments each resulting from different embryological veins (from cranial to caudal): *suprahepatic segment* (right vitelline vein), *hepatic segment* (right vitelline-*sub*cardinal anastomosis), *suprarenal segment* (right *sub*cardinal vein), *renal segment* (right sub-supracardinal anastomosis), *infrarenal segment* (right *supra*cardinal vein), *terminal IVC* and *common iliac veins* (posterior cardinal veins) (Fig. 2b). Most inferior caval abnormalities result of abnormal development of these segments, nevertheless, even in severe malformation cases, some form of venous flow exist by replacement from another of the constituent embryologic networks [1, 2].

#### Anomalies

**Fig. 6:**
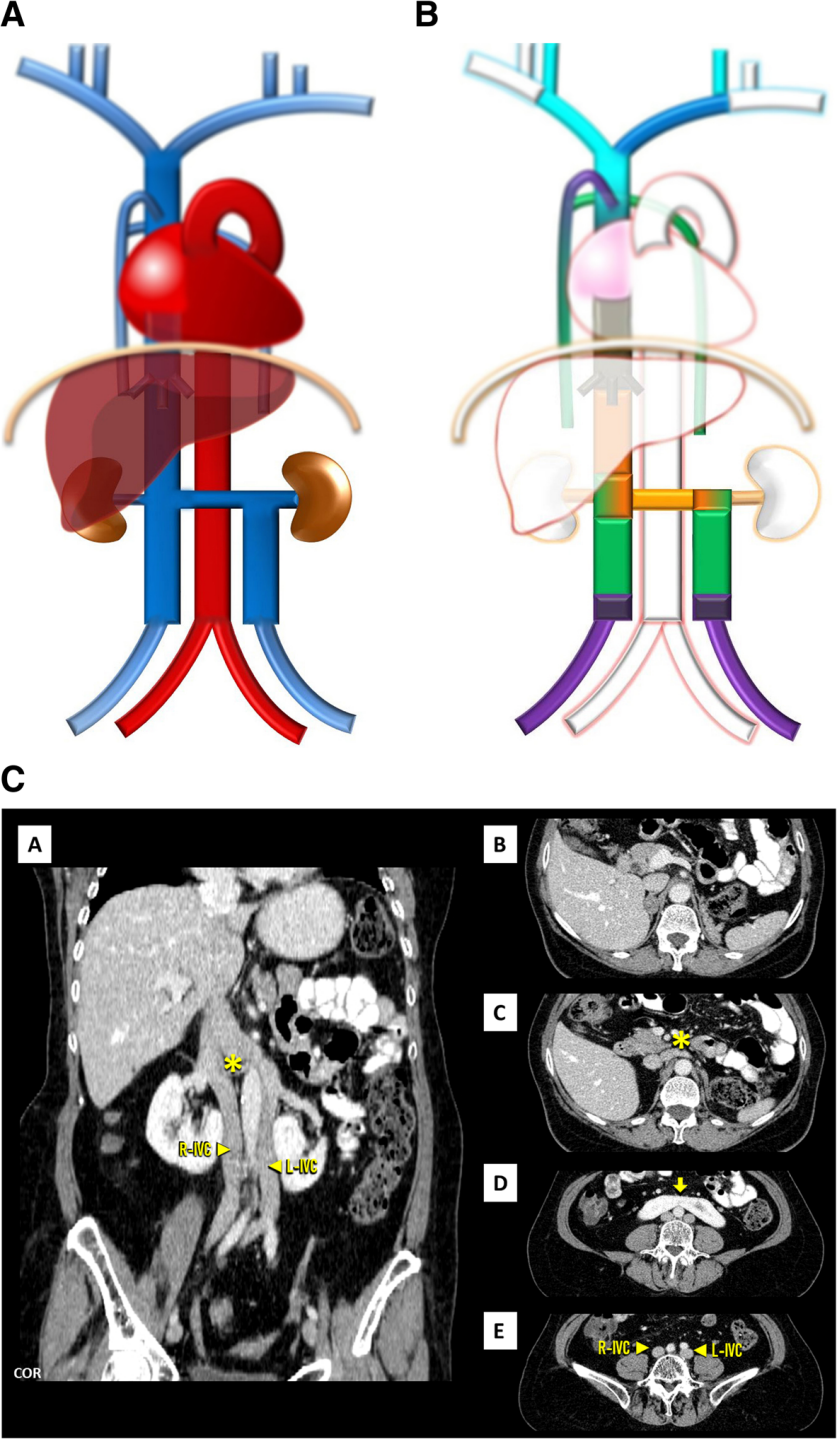
**a** Double IVC schematic. With the left component draining to the left renal vein, which joins the right IVC as normal. **b** Double IVC embryology schematic. Double IVC resulting from the persistence of both *supra*cardinal veins, uniting in a single trunk on the right through the *sub-supra* and inter-*sub*cardinal anastomosis, forming the left renal vein. **c** Double IVC imaging. Contrast-enhanced CT showing duplicated IVC, with a normal right-sided IVC (R-IVC) and a left-sided IVC (L-IVC) which ends at the left renal vein (asterisk) [C], note also horseshoe kidney (arrow) [D]

Duplicated IVC (Fig. 6a–c): As the result from the persistence of both *supra*cardinal veins is one of the most prevalent abnormality, with a prevalence of about 0.2–3% [3]. Typically, the left component drains to the left renal vein, which joins the right IVC as normal. There may be, however, variations in this arrangement and size asymmetry between the left and right veins. Clinically, it should be suspected if recurrent pulmonary thromboembolism occurs, in spite of placement of an IVC filter [4].

**Fig. 7:**
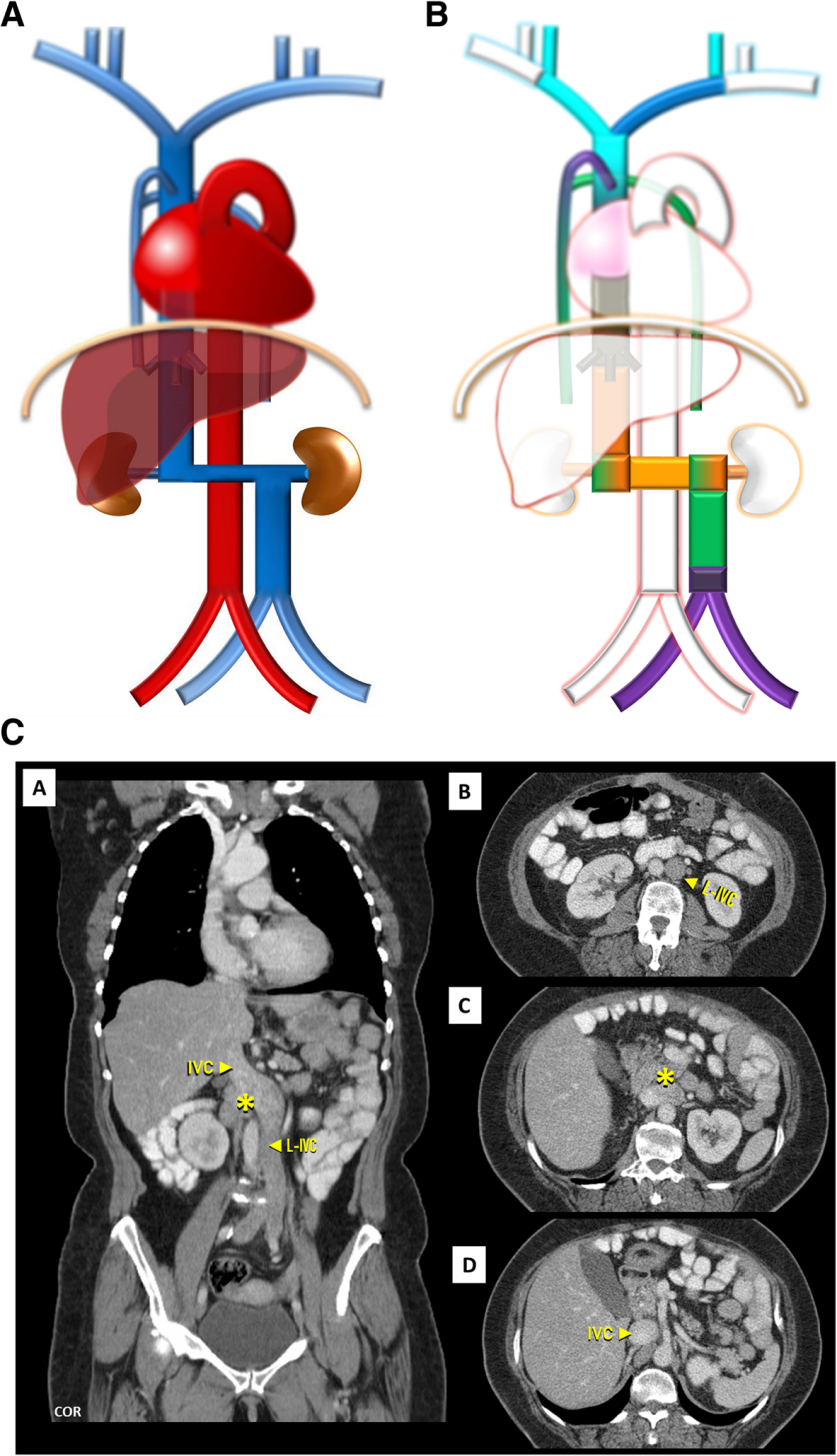
**a** Left-sided IVC schematic. Being different from duplication as the infrarenal segment of the right IVC is absent .The left IVC joins the left renal vein, which in normal fashion crosses ventrally the aorta to join the right renal vein from which results a normal suprarenal segment of the IVC. **b** Left-sided IVC embryology schematic. Left-sided IVC resulting from the regression of the right *supra*cardinal vein, in addition to the persistence of the left *supra*cardinal vein. **c** Left-sided IVC imaging. Contrast-enhanced CT showing a left-sided IVC (L-IVC), which characteristically joins the left renal vein, who in a normal fashion crosses ventrally to join the right renal vein (asterisk) and form a normal suprarenal segment of the IVC

Left-sided IVC (Fig. 7a–c): Resulting of the persistence of the left *supra*cardinal vein, in addition to the regression of the one on the right, has a prevalence of about 0.2–0.5% [3]. Usually, as above, the left IVC joins the left renal vein, which in normal fashion crosses ventrally to join the right renal vein, which results in a normal suprarenal segment of the IVC. It may complicate placement of an IVC filter on the infrarenal segment via a transjugular access [4].

**Fig. 8:**
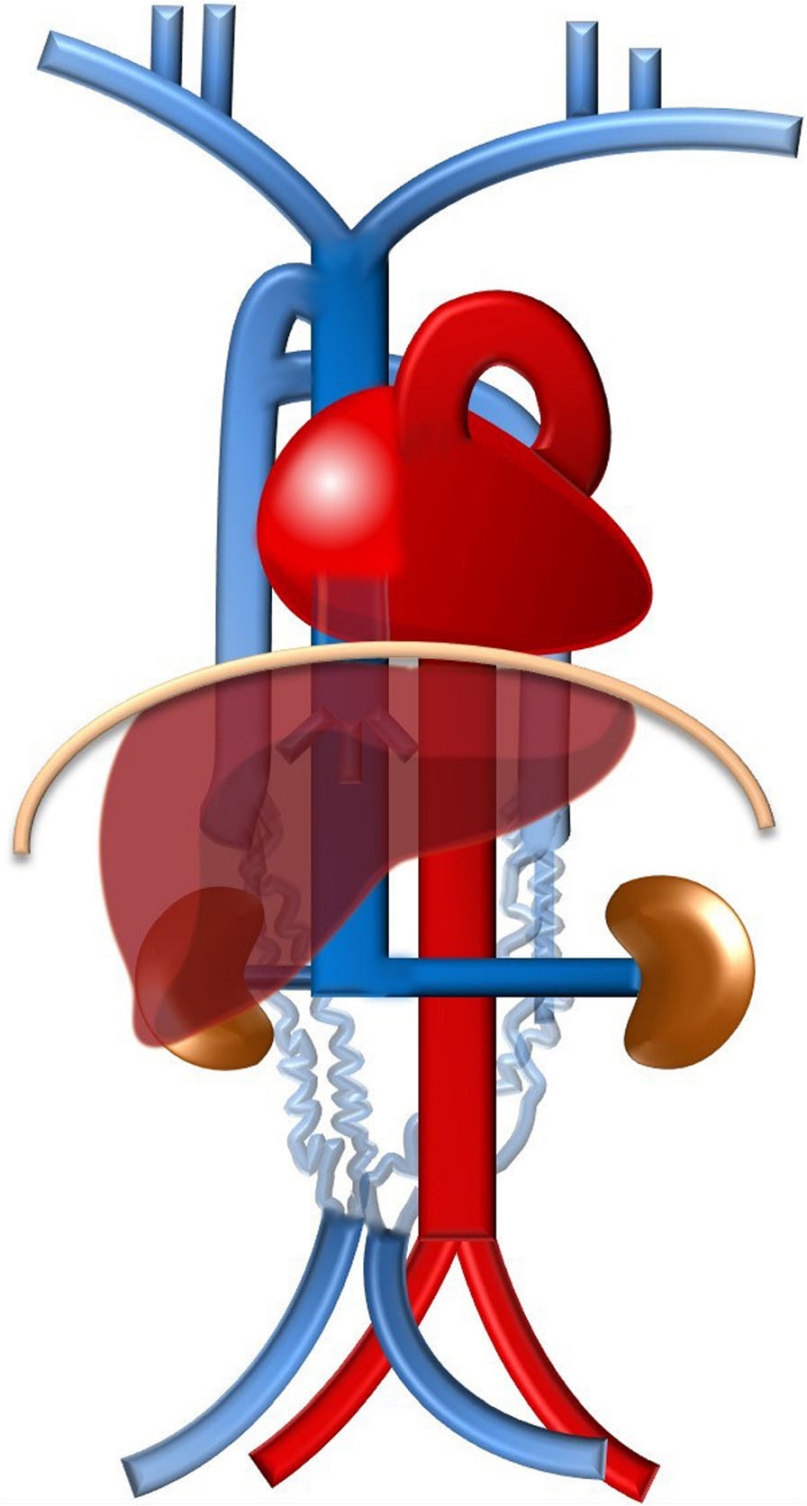
Interruption of the infrarenal IVC with preservation of the suprarenal segment schematic. A rarer anomaly, subsequent to abnormal embryologic development of the *posterior* cardinal and supracardinal veins with resulting absence of the infrarenal segment of the IVC, in which the blood from the lower half of the body reaches the heart via the azygo-hemiazygos system of veins and prominent collateral vessels

Interruption of the infrarenal IVC with preservation of the suprarenal segment (Fig. 8): A rarer anomaly, subsequent to abnormal embryologic development of the *posterior* cardinal and *supra*cardinal veins with resulting absence of the infrarenal segment of the IVC. Blood from the lower half of the body reaches the heart via the SVC by means of the azygos-hemiazygos system, subsequently dilated to the increased flow, which receives blood from the caudal part of the body via collateral drainage by ascending lumbar veins. A normal suprarenal IVC is formed by the confluence of the renal veins. Clinically, it may result in chronic lower-extremity venous insufficiency and deep venous thrombosis [4].

**Fig. 9:**
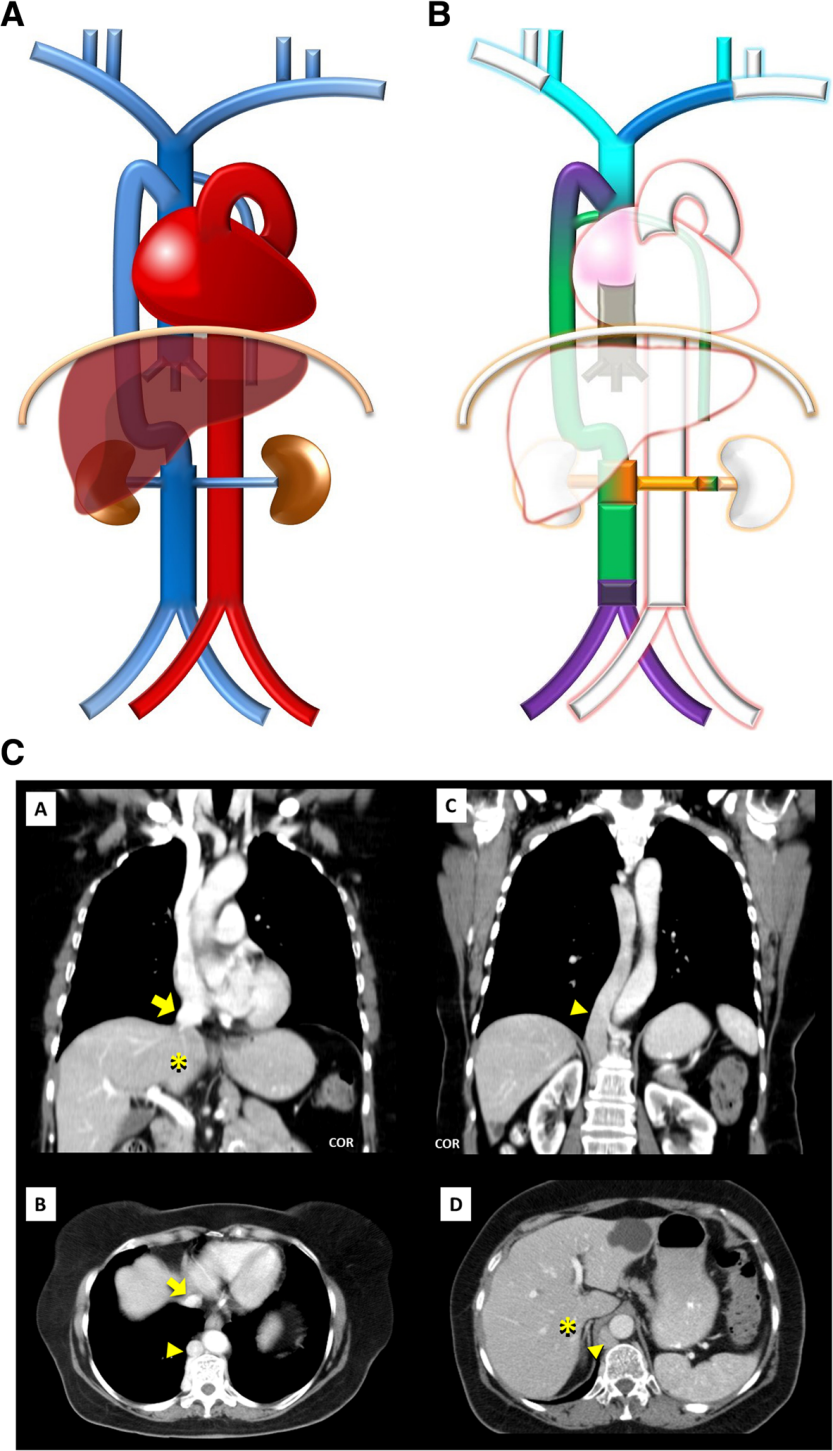
**a** Interruption of the hepatic segment of the IVC/Azygos continuation of the IVC schematic. In this anomaly, the hepatic segment of the IVC is absent or hypoplastic, with the hepatic veins draining into the right atrium via the *supra*hepatic IVC and the renal portion of the IVC, receiving the blood return from the kidneys and lower extremities, by means of the azygos vein. **b** Interruption of the hepatic segment of the IVC/Azigos continuation of the IVC embryology schematic. The result from the failure to form of the right vitelline-*sub*cardinal anastomosis, with consequential regression of the right *sub*cardinal vein, causing the blood to directly shunt into the right *supra*cardinal vein. **c** Interruption of the hepatic segment of the IVC/Azygos continuation of the IVC imaging. Contrast-enhanced CT scan denoting absence of the hepatic segment of the IVC (asterisk), drainage of the hepatic veins into the right atrium via the suprahepatic IVC [A, B] and continuation of the renal portion of the IVC, passing posterior to the diaphragmatic crura to enter the thorax as the azygos vein (triangle), which drains to the SVC as normal [C, D]

Interruption of the hepatic segment of the IVC/Azygos continuation of the IVC (Fig. 9a–c): Resulting from the failure to form of the right vitelline-*sub*cardinal anastomosis, with consequential regression of the right *sub*cardinal vein, causing the blood to directly shunt into the right *supra*cardinal (Fig. 9b). It has a prevalence of about 0.6% but may reach 1.3% when concomitant congenital heart disease, asplenia or polysplenia syndromes (left isomerism) [4, 5]. As a result, the hepatic segment is absent or hypoplastic, with the hepatic veins draining into the right atrium via the suprahepatic IVC. The renal portion of the IVC, receiving the blood return from the kidneys and lower extremities, empties to the SVC by means of the azygos vein, with the azygos vein, azygos arch, and SVC being subsequently dilated to the increased flow. Preoperative knowledge of this anatomic arrangement is imperative in percutaneous cardiopulmonary procedures [4, 5].

**Fig. 10:**
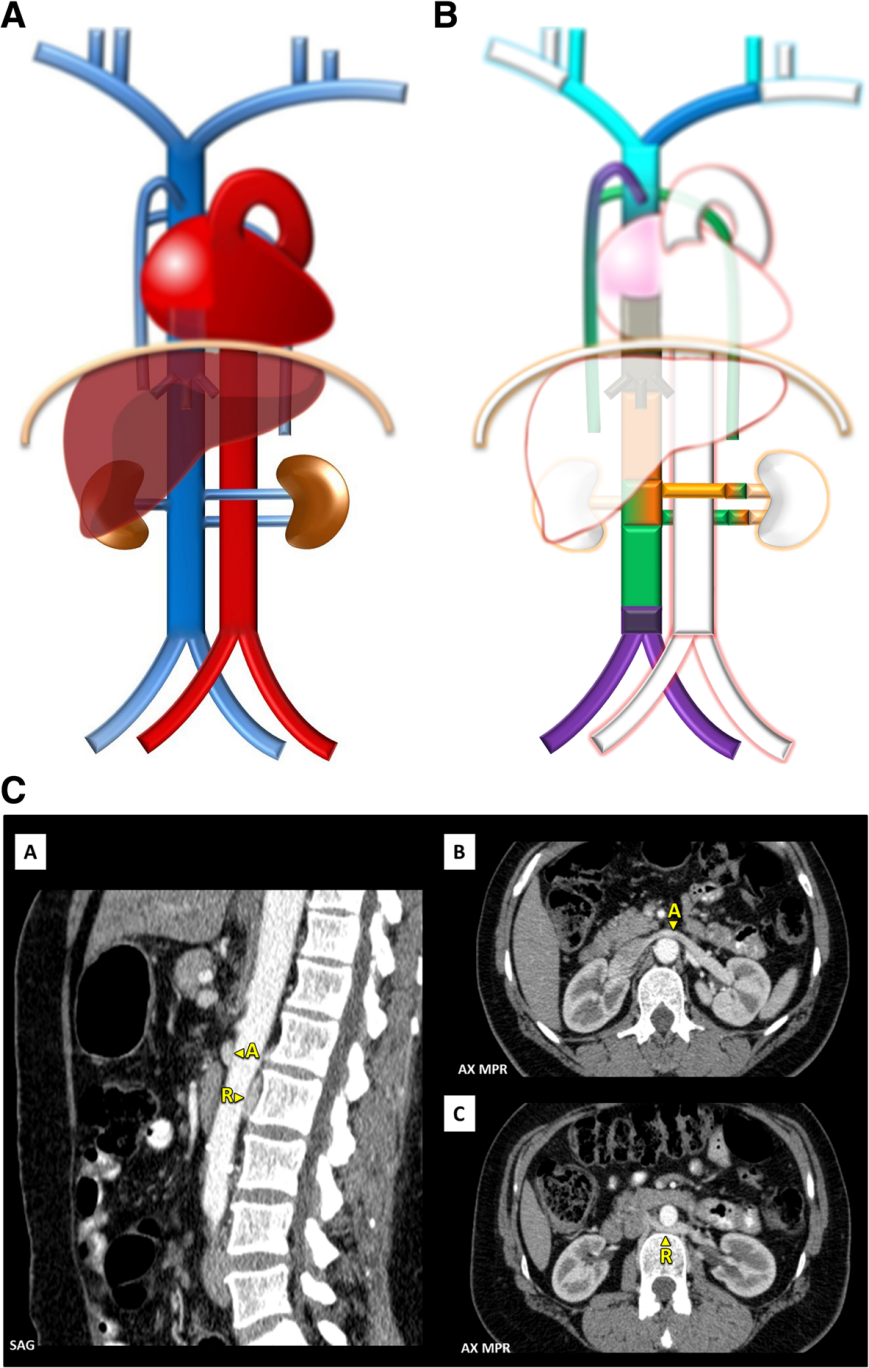
**a** Circumaortic left renal vein schematic. In the case of the existence of two left renal veins, one superior, that crosses ventral to the aorta, and one inferior, that crosses dorsal to the aorta. **b** Circumaortic left renal vein embryology schematic. Showing the persistence of both venous arches of the left renal vein, one superior and ventral to the aorta, composed by the inter-*sub*cardinal anastomosis and ventral limbs of the left *sub-supra*cardinal anastomosis and left renal vein, and one inferior and dorsal to the aorta, composed by the inter-*supra*cardinal anastomosis, and dorsal limbs of the left *sub-supra* cardinal anastomosis and left renal vein. **c** Circumaortic left renal vein imaging. Contrast-enhanced CT presentation of a circumaortic left renal vein with an anterior left renal vein (A) and a retro-aortic left renal vein (R)

**Fig. 11:**
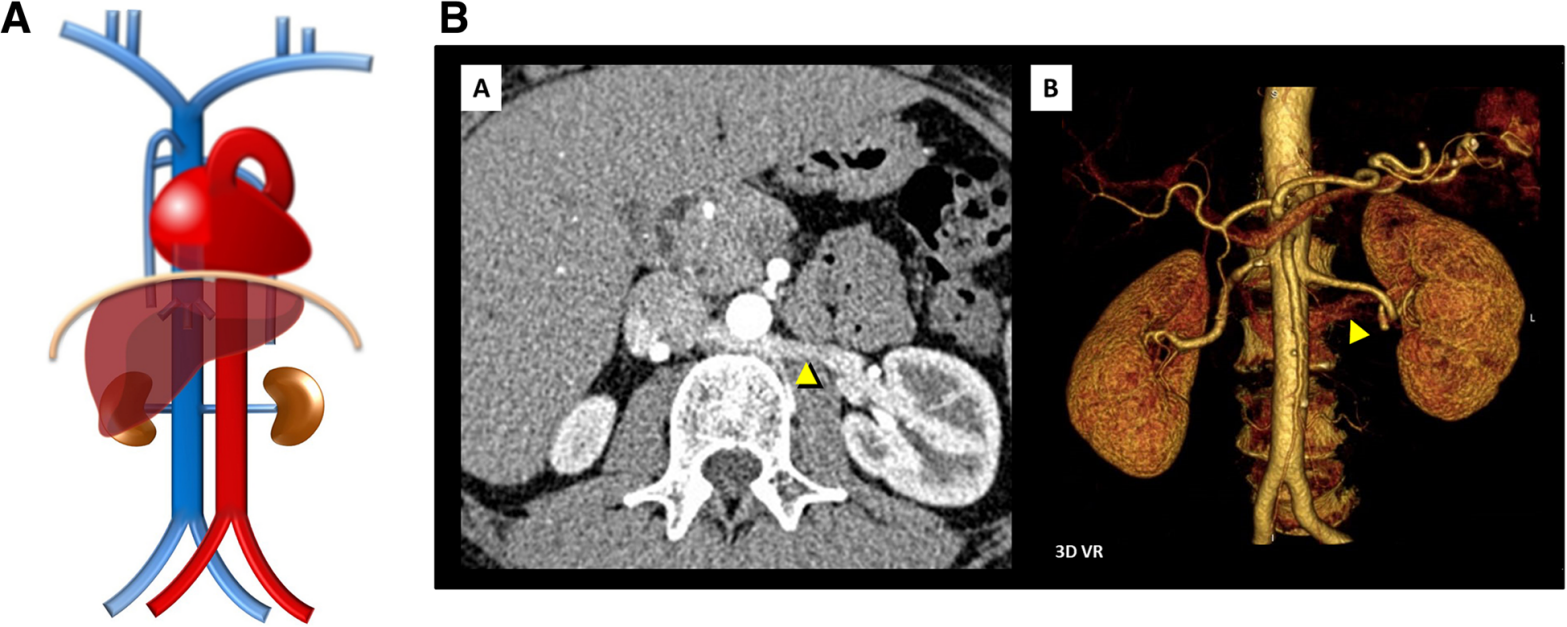
**a** Retroaortic left renal vein schematic. Contrasting as there is a single renal vein passing dorsal to the aorta. **b** Retroaortic left renal vein. As shown on contrast-enhanced CT scan [A] and on volumetric 3D reconstruction [B]

Left renal vein associated anomalies: Are the most common abnormalities and result from variations in the persistence and regression of the two components of the embryonic left renal vein collar, consisting of a ventral arch, an inter-*sub* cardinal anastomosis, and a dorsal arch, an inter-*supra* cardinal anastomosis, and respective limb of the embryonic left renal vein, the second of which normally regresses (Fig. 10b). Knowledge of this anatomy may be important prior to renal vein catheterization and on the preoperative planning for nephrectomy [4].In a circumaortic left renal vein (Fig. 10a/c), whose prevalence may be as high as 8.7%, the persistence of both archs results in the presence of two left renal veins: one superior that receives the left suprarenal vein and crosses ventral to the aorta, usually at the same level as its origin at the hilum, and one inferior, usually longer, that receives the left gonadal vein and crosses dorsal to the aorta, each draining into the IVC via different openings.In a retroaortic left renal vein (Fig. 11a/b), present in about 2%, the persistence of the dorsal arch is associated with regression of the ventral arch (inter-*sub* cardinal anastomosis) resulting in a single renal vein passing dorsal to the aorta [4].

**Fig. 12:**
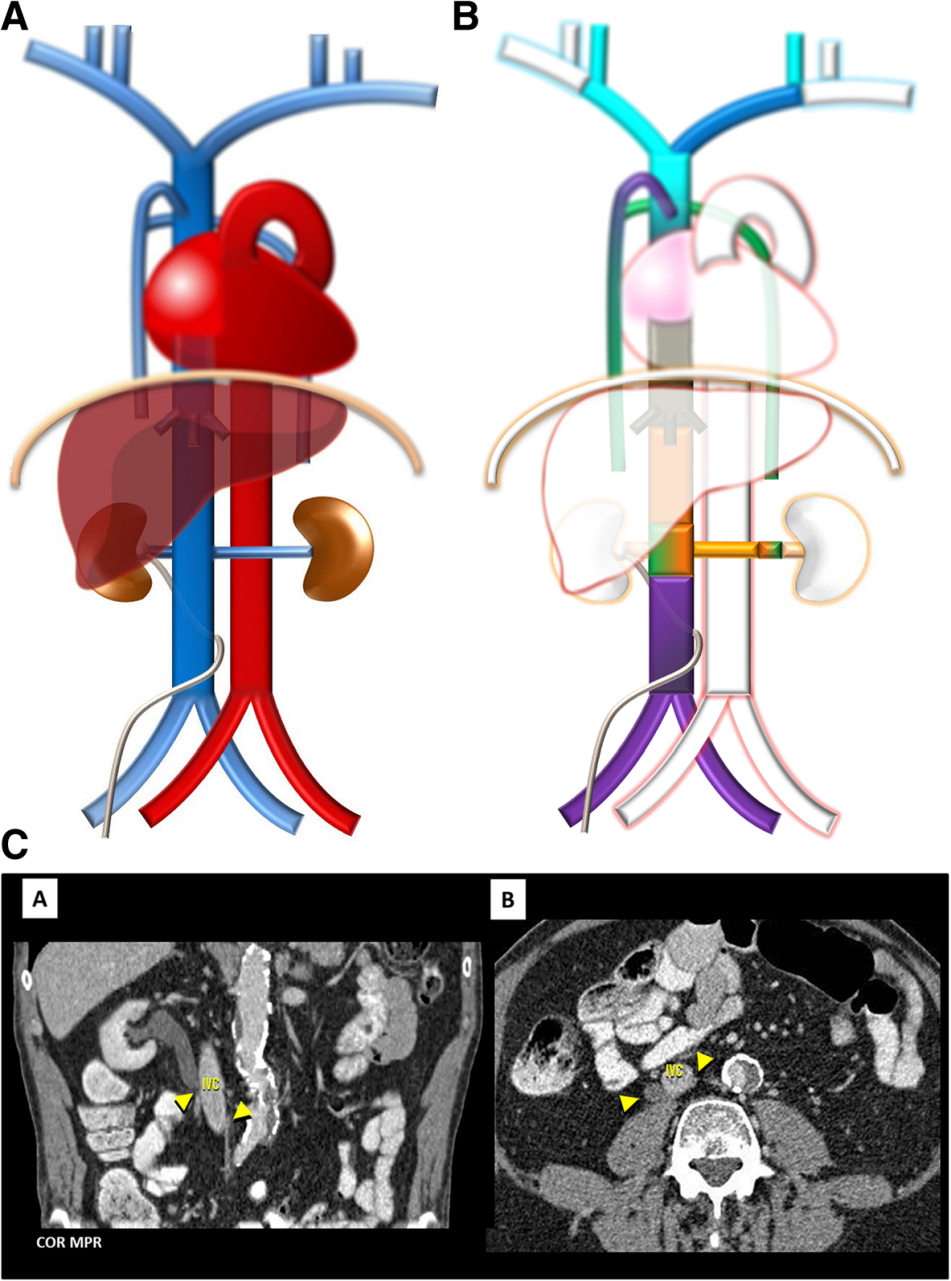
**a** Circumcaval ureter schematic. An anomaly as the name implies, in which the ureter begins to course dorsal to the IVC, then arches the IVC to come to lie ventrally. **b** Circumcaval ureter embryology schematic. Resulting from the persistence of the right posterior cardinal vein positioned ventral to ureter in the definitive IVC. **c** Circumcaval ureter imaging. Contrast-enhanced CT presentation of a circumcaval ureter, which begins to course dorsal to the IVC, then arches the IVC by the left, coming to lie anteriorly, associated with mild proximal ureterohydronephrosis

Circumcaval ureter (Fig. 12a–c): Result from an anomaly of the right *supra*cardinal system with persistence of the right *posterior* cardinal vein positioned ventral to ureter in the definitive IVC, occurring in about 0.07% [4]. The ureter begins to course dorsal to the IVC, then arches the IVC by the left, coming to lie anterior to the right common iliac vein. This can lead to some degree of ureteral obstruction (Fig. 12c) and recurrent urinary tract infections due to urinary stasis, although many patients are asymptomatic.

**Fig. 13:**
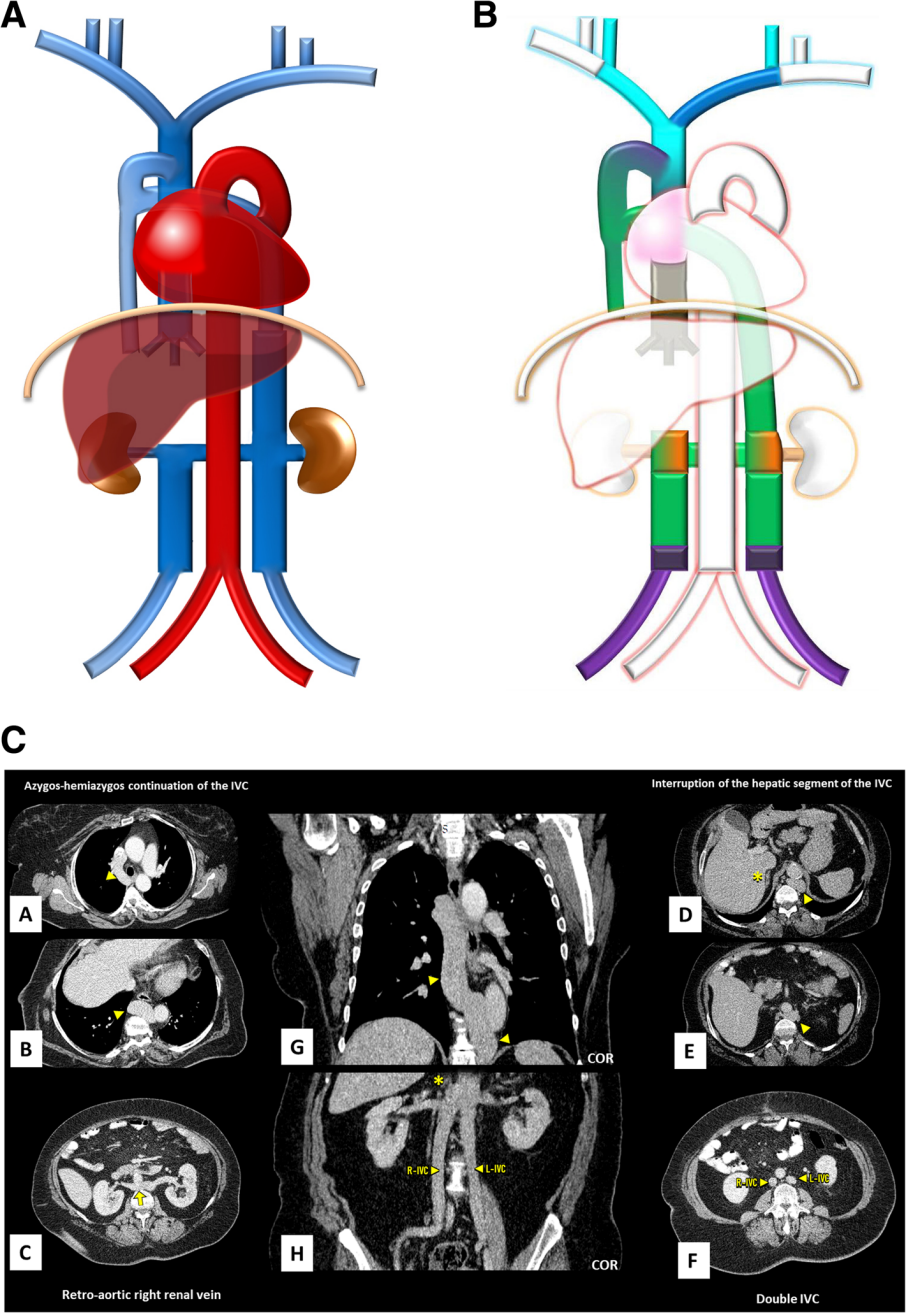
**a** Coexistence of multiple venous development anomalies schematic. Case of a double IVC with a retro-aortic right renal vein, interruption of the hepatic segment and hemiazygos continuation of the IVC. **b** Coexistence of multiple venous development anomalies embryology schematic. Resulting from the persistence of the left *supra*cardinal veins and the left sub-supracardinal anastomosis, regression of the right vitelline-*sub*cardinal anastomosis, together with persistence of the dorsal arch and regression of the ventral arch of the renal collar. **c** Coexistence of multiple venous development anomalies imaging. Contrast-enhanced CT scan showing a double IVC (R-IVC, L-IVC) [F] with a retroaortic right renal vein (arrow) [C], interruption of the hepatic segment (asterisk) [D, H] and hemiazygos continuation of the IVC (triangle) [A, B, E]

More than one anomaly can coexist, as such the case of a duplicated IVC with retroaortic right renal vein, interruption of the hepatic segment and hemiazygos continuation of the IVC, resulting from the persistence of the left *supra*cardinal veins and the left *sub-supra*cardinal anastomosis, regression of the right vitelline-*sub*cardinal anastomosis, together with persistence of the dorsal arch and regression of the ventral arch of the renal collar (Fig. 13a–c).

### Azygos-hemiazygos system associated anomalies

#### Anatomy

The azygos-hemiazygos system is a paravertebral venous pathway located in the posterior mediastinum, comprised of the azygos, hemiazygos and accessory hemiazygos veins. It receives the venous blood of the thoracic wall and superior lumbar region by means of the lumbar and posterior intercostal veins [9].

The azygos vein is a vessel that arises from the confluence of the right ascending lumbar vein and the right subcostal vein around T12-L2. It enters the thorax passing through the diaphragm via the aortic hiatus and then ascends along the right anterolateral surface of the thoracic vertebrae, to end on the SVC at the level of T4, after arching over the right main bronchus, in what is called the azygos arch, which contains a valve halfway which may lead to accumulation of the intravenously injected contrast agent on imaging studies. Although much variability exists, the azygos vein usually receives as collaterals the accessory hemiazygos vein and the hemiazygos vein at the level of T8 and T9, respectively.

The hemiazygos vein is a similar structure on the opposite side of the vertebral column and analogously is formed by the confluence of the left ascending lumbar and left subcostal veins. It enters the thorax either through the aortic hiatus or directly through the diaphragmatic crura, ascends along the left anterolateral aspect of the thoracic vertebrae and at T8-T9 crosses behind the descending thoracic aorta to drain into the azygos vein.

The accessory hemiazygos vein which drains the superior left hemithorax and left bronchial vein arises from the fourth to eighth left posterior intercostal veins, as a left paravertebral course and joins the azygos vein behind the esophagus at different levels, usually at the level of T8.

The bilateral superior intercostal veins drain the second to fourth intercostal spaces and join respectively the azygos vein and the left brachiocephalic vein.

#### Embryogenesis

The azygos-hemiazygos venous system arises from the *supra*cardinal veins embryologically (Fig. 2): the azygos vein considered to derive from the upper right *supra*cardinal vein, the hemiazygos vein from the upper left *supra*cardinal vein and the arch of the azygos from an upper segment of the right *posterior* cardinal vein [1, 2]. In the abdominal cavity, the *supra*cardinal veins become the infrarenal portion of the definitive inferior vena, as thus communication between both systems is possible. Variants of the azygos-hemiazygos system anatomy are frequent, particularly on the arrangement of the hemiazygos and accessory hemiazygos veins, which can join on a common trunk instead of draining individually. Variations may also be acquired, due to collateralization of blood flow in cases of SVC or IVC obstruction.

#### Anomalies

**Fig. 14:**
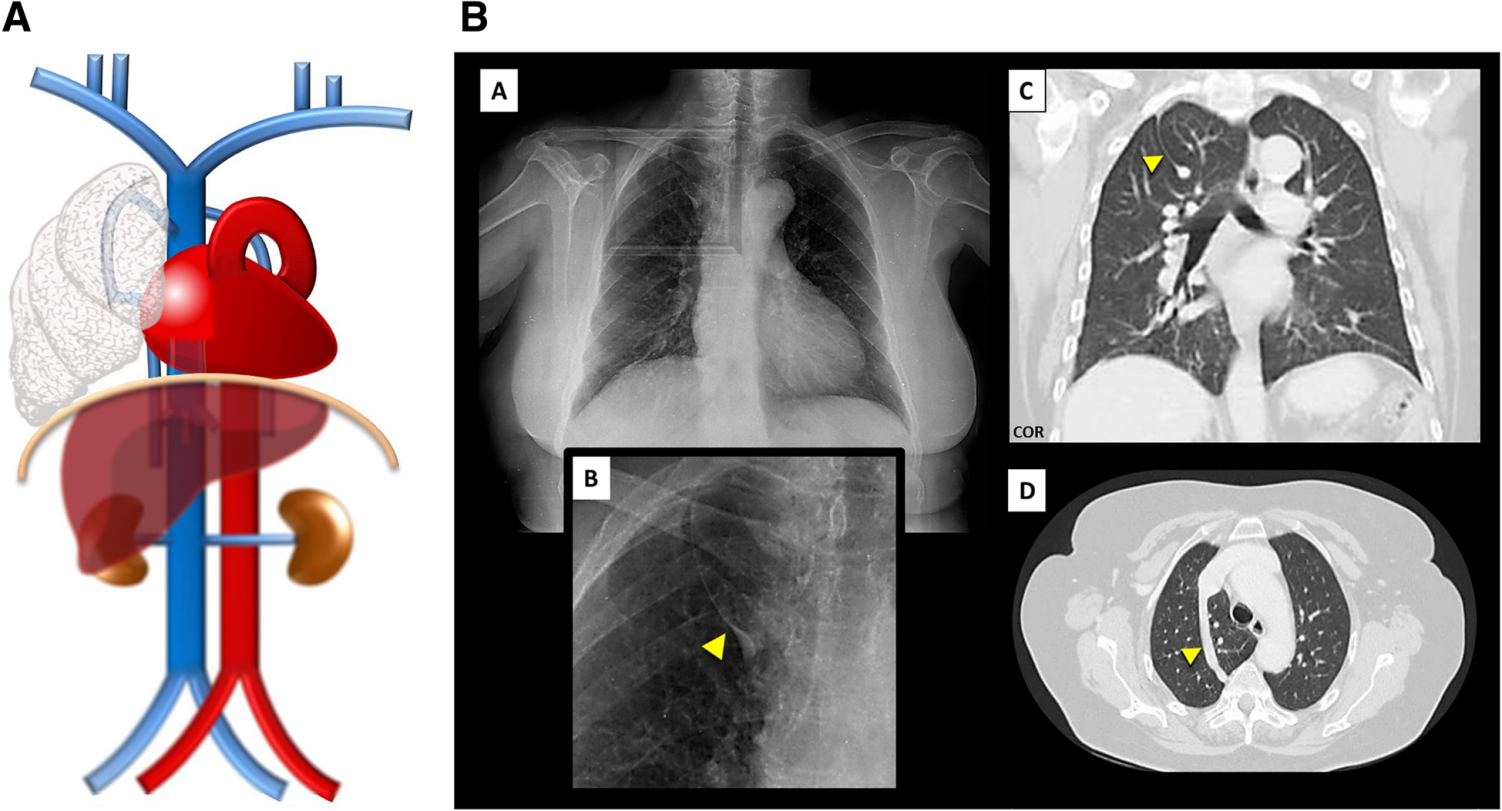
**a** Azygos fissure and lobe schematic. Resulting from an abnormal course of the azygos vein in the apex of the right lung subsequent to a failure of migration. **b** Azygos fissure and lobe imaging. Azygos fissure and lobe, as seen on chest X-ray [A, B—magnification view], as a line that crosses the apex of the right lung ending in a tear shape, and on thoracic CT scan on coronal [C] and axial [D] planes

**Fig. 15:**
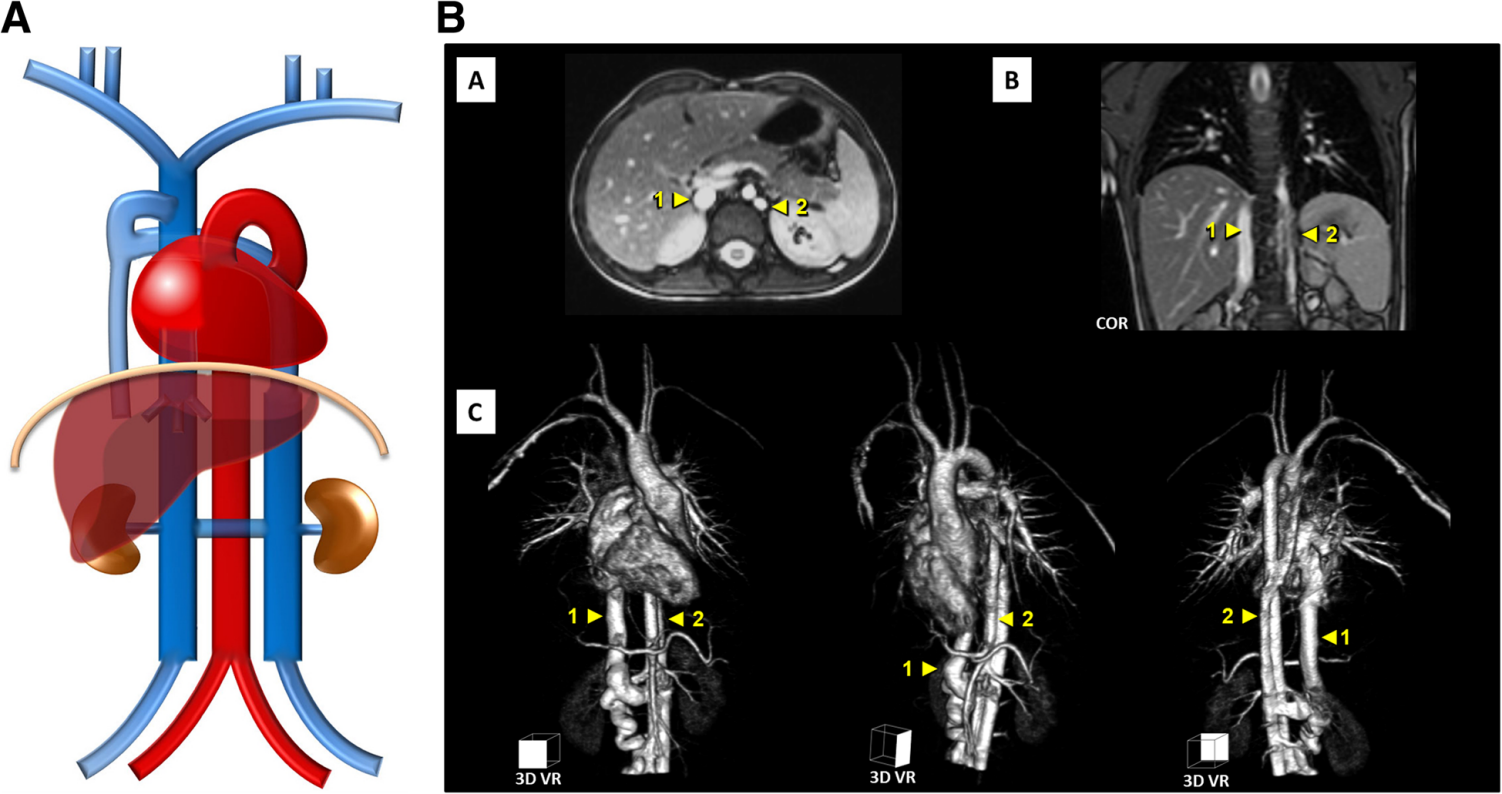
**a** Double IVC with hemiazygos continuation of the IVC schematic. Resulting from the persistence of the in-between segment of the supracardinal vein that connects both the IVC and the azygos-hemiazygos system. **b** Double IVC with hemiazygos continuation of the IVC imaging. MR angiography [A—axial plane, B—coronal plane, C—volumetric 3D reconstruction] showing double IVC with hemiazygos continuation of the left-sided IVC, in which (“1”)—normal right-sided IVC and (“2”)—hemiazygos continuation of the left-sided IVC

Azygos fissure and lobe (Fig. 14a/b): An anomaly found in approximately 0.4–1% of the population resulting from an abnormal course of the azygos vein in the apex of the right lung [3] subsequent to a failure of migration of the *posterior* cardinal vein. The abnormally laterally located vein indents the lung and both parietal and visceral pulmonary pleurae, resulting in the azygos mesofissure, a fissure with four pleural layers, similarly to a mesentery structure [10]. It is easily identified on imaging studies, including conventional chest radiographs, where it presents a tear drop shape (Fig. 15c). The azygos fissure may isolate pathological processes developing in and from the rest of the lung tissue, and so, for example, carcinoma arising on the azygos lobe was shown not to be commonly associated with regional lymph node involvement [11].

Azygos/hemiazygos continuation of the IVC: Normally, the in-between segment of the supracardinal vein that connects both the IVC and the azygos-hemiazygos system regresses, but if the suprarenal segment of the IVC fails to develop, it may persist, resulting in azygos or hemiazygos continuation (as seen on Fig. 15a/b and previously on Figs. 9 and 13). As previously cautioned, knowledge of this anatomic arrangement is important in percutaneous cardiopulmonary procedures.

**Fig. 16:**
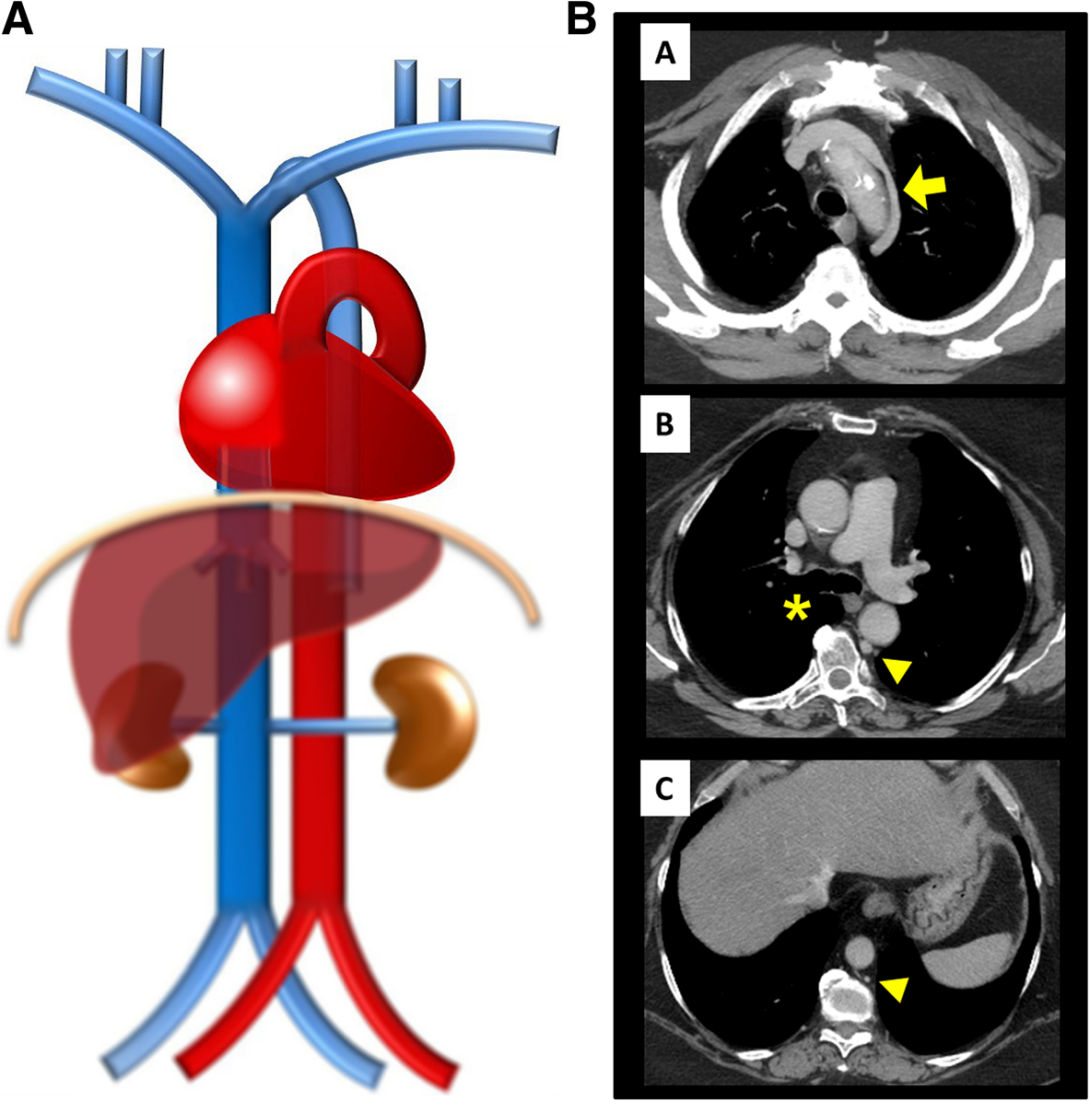
**a** Absent azygos vein schematic. Congenital absence of the azygos vein is associated with enlargement of the remaining paravertebral vessels, as the hemiazygos, that serve as a collateral pathway draining usually into the left brachiocephphalic vein. **b** Absent azygos vein imaging. Contrast-enhanced CT scan denoting an absent azygos vein (asterisk) with enlargement of the hemiazygos (triangle) which drains to the left brachiocephalic vein by an enlarged left superior intercostal vein (arrow)

Absent azygos vein (Fig. 16a/b): Congenital absence of the azygos vein is rare and associated with enlargement of remaining paravertebral vessels, that serve as a collateral pathway draining usually into the left brachiocephphalic vein.

**Fig. 17:**
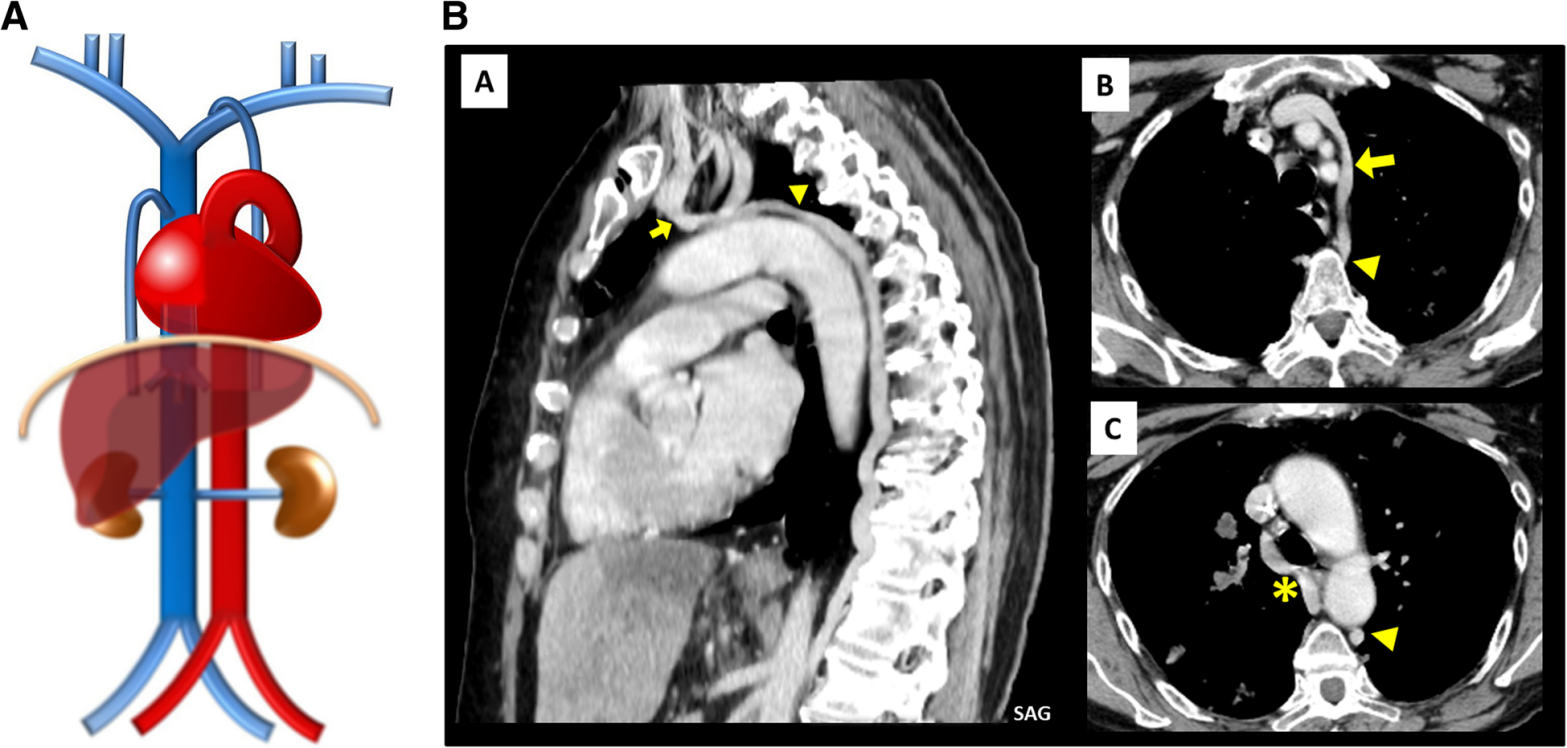
**a** Variable hemiazygos drainage schematic. Independent drainage of the hemiazygos vein to the SVC, via the accessory hemiazygos vein→left superior intercostal vein→left brachiocephphalic vein path. **b** Variable hemiazygos drainage imaging. Contrast-enhanced CT scan showing independent drainage of the hemiazygos vein (triangle) to the left brachiocephphalic vein, via the left superior intercostal vein (arrow), with normal drainage of the azygos vein via the azygos arch (asterisk)

Variable hemiazygos drainage (Fig. 17a/b): Usually, the hemiazygos vein drains to the azygos vein but could also drain to the right SVC, via the accessory hemiazygos vein→left superior intercostal vein→left brachiocephphalic vein path, or to a persistent left SVC. This often occurs with duplicated IVCs, and the different drainage routes will influence the imaging findings of a hemiazygos continuation of a left-sided IVC and may result in a rarer hemiazygos lobe [3].

The azygos vein may itself also rarely not drain into de SVC but into the right brachiocephalic or subclavian vein or directly into the right atrium [10, 12].

These variants of drainage of the azygos-hemiazygos system have no clinical significance.

## Conclusion

Congenital anomalies of the systemic venous return although rare are incidentally found, and so radiologists must knowledge and be aware of the normal venous anatomy and most common anatomic variants, primarily to avoid diagnostic pitfalls, as well as some of these conditions can have technical implications on invasive procedures and in a preoperative setting [13].

## Data Availability

Not applicable

## Abbreviations

CT: Computed tomography
IVC: Inferior vena cava
MR: Magnetic resonance
SVC: Superior vena cava

## References

[1] Pansky B (1982) Review of MEDICAL EMBRYOLOGY. Embryome Sciences Inc, Alameda, CA

[2] Franziska Schöni-Affolter (2016) Organogenesis: chapter 16.6 - development of the veins - University of Fribourg; Available via http://www.embryology.ch/anglais/pcardio/venen01.html#Kardinales; Accessed 22 December 2017.

[3] Demos TC, Posniak HV, Pierce KL, Olson MC, Muscato M (2004) Venous anomalies of the thorax. AJR Am J Roentgenol 82(5):1139–1150 10.2214/ajr.182.5.1821139 15100109

[4] Bass JE, Redwine MD, Kramer LA (2000). (2000), Spectrum of congenital anomalies of the inferior vena cava: cross-sectional imaging findings. Radiographics.

[5] Mazzucco A, Bortolotti U, Stellin G, Galucci V (1990). Anomalies of the systemic venous return: a review. J Card Surg.

[6] Cormier MG, Yedlicka JW, Gray RJ, Moncada R (1989). Congenital anomalies of the superior vena cava: a CT study. Semin Roentgenol 24:77–83 PMID:2727725 DOI:10.1016/0037-198X(89)90028-X 10.1016/0037-198x(89)90028-x 2727725

[7] HHapugoda S, Bashir O et al. Superior vena cava. Avaliable via https://radiopaedia.org/articles/superior-vena-cava; Accessed 22 December 2017.

[8] Carrol D, Jones J et al. Inferior vena cava. Available via https://radiopaedia.org/articles/inferior-vena-cava; Accessed 22 December 2017.

[9] Robertson S, Knipe H et al. Azygos venous system. Available via https://radiopaedia.org/articles/azygos-venous-system; Accessed 22 December 2017.

[10] Dudiak CM, Olson MC, Posniak HV (1991) CT evaluation of congenital and acquired abnormalities of the azygos system. Radiographics 11(2):233–246. 10.1148/radiographics.11.2.2028062 10.1148/radiographics.11.2.2028062 2028062

[11] Kotov G, Dimitrova IN, Iliev A, Groudeva V (2018). A rare case of an azygos lobe in the right lung of a 40-year-old male. Cureus.

[12] Cole TJ, Henry DA, Jolles H, Proto AV (1995) Normal and abnormal vascular structures that simulate neoplasms on chest radiographs: clues to the diagnosis. Radiographics 15:867–891. 7569133. 10.1148/radiographics.15.4.7569133 10.1148/radiographics.15.4.7569133 7569133

[13] Oliveira JD, Martins I (2018). ECR 2018/C-2260: All roads lead to R..ight atrium: general review of congenital anomalies of the systemic venous return.

